# Seasonal variation of cardiac structure and function in the elite rugby football league athlete

**DOI:** 10.1186/s44156-023-00026-9

**Published:** 2023-10-11

**Authors:** Lynsey Forsythe, Keith George, Michael Papadakis, Nathan Mill, Matt Daniels, David Oxborough

**Affiliations:** 1 University Hospitals Bristol and Weston NHS Foundation Trust, Bristol, UK; 2 Research Institute for Sport and Exercise Sciences, Liverpool John Moores University, Tom Reilly Building, Liverpool, L3 3AF UK; 3 Cardiovascular Sciences Research Centre, St Georges University of London, London, UK; 4 St Helens Rugby Football League Club, St Helens, UK

**Keywords:** Athlete’s heart, Seasonal variation, Cardiac screening, Echocardiography, Speckle tracking echocardiography

## Abstract

**Background:**

Pre-participation cardiac screening (PCS) of “Super-League” rugby football league (RFL) athletes is mandatory but may be completed at any time point. The aim of this study was to assess cardiac electrical, structural and functional variation across the competitive season.

**Methods:**

Elite, male, RFL athletes from a single Super-League club underwent cardiac evaluation using electrocardiography (ECG), 2D echocardiography and speckle tracking echocardiography (STE) at four time points across the RFL season; (1) End pre-season (ENDPRE), (2) mid-season (MIDCOMP), (3) end-season (ENDCOMP) and (4) End off-season (ENDOFF). Training loads for each time point were also determined. One-way ANOVA with post-hoc Bonferroni were used for statistical analyses.

**Results:**

Total workload undertaken by athletes was lower at both MIDCOMP and ENDCOMP compared to ENDPRE (P < 0.001). ECG patterns were normal with training-related changes that were largely consistent across assessments. Structural data did not vary across assessment points. Standard functional data was not different across assessment points but apical rotation and twist were higher at ENDPRE (9.83˚ and 16.55˚, respectively compared to all other time points (MIDCOMP, 6.13˚ and 12.62˚; ENDCOMP, 5.84˚ and 12.12˚; ENDOFF 6.60˚ and 12.35˚).

**Conclusions:**

Despite some seasonal variation in training load, the athletes’ ECG and cardiac structure were stable across a competitive season. Seasonal variation in left ventricular (LV) apical rotation and twist, associated with higher training loads, should be noted in the context of PCS.

## Background

Pre-participation cardiac screening (PCS) is undertaken to reduce the risk of sudden cardiac death (SCD) by identifying, or excluding, an underlying inherited cardiac condition. Knowledge of cardiac adaptation to training, including electrical, structural and functional changes [1–7], referred to as the athletes’ heart (AH) is vitally important to differentiate the AH from pathology and aid PCS. Despite the malleability of the AH with variation in training loads, there are no specific guidelines as to when PCS should occur during a competitive season in athletes.

The limited electrocardiographic (ECG) or echocardiographic studies undertaken across various stages of a competitive season are often contradictory [3, 8–13]. An early study demonstrated augmented cardiac adaptation in cyclists during the season with decreased left ventricular (LV) wall thickness and a slight decrease in LV function seen in the off-season [9]. Other studies have demonstrated an increased LV mass in male soccer players within the competitive season with concomitant regression reported with detraining [3, 14]. Disparate findings may be related to the lack of clear documentation of workload variation and different sporting disciplines. Whilst increased atrial size is considered a normal physiological response to exercise training [15] it may mimic other pathological causes [16]. Changes in left atrial (LA) volumes have been associated with changes in LA morphology during adaptation to training in soccer players [17]. Seasonal studies involving myocardial strain (ɛ) imaging by speckle tracking echocardiography (STE) are equally sparse [12, 18–20] and therefore, further assessment of seasonal variation in cardiac electrical, structural and functional indices is warranted.

Rugby football league (RFL) is an intermittent sport incorporating moderate static and moderate dynamic activity [21]. PCS is mandatory for athletes competing in the RFL Super-League with previous cross sectional studies highlighting the nature and extent of left and right heart remodelling of RFL athletes’ heart compared to controls [22, 23]. The RFL competitive season runs over 9 months with defined variations in training type and intensity throughout this period. These athletes may present for PCS at any time during the competitive season therefore, forming the rationale of this study to assess the impact of seasonal variation on cardiac structure and function. Consequently, the primary aim of this study was to assess electrical, structural and functional cardiac data in RFL athletes at clearly defined stages of the season using ECG, standard 2D echocardiography and STE.

## Methods

### Study population and design

Following ethical approval, elite, senior, male RFL athletes, representing multiple ethnic groups, were recruited from a single Super-League club during mandatory PCS. Athletes provided full written informed consent to participate in the study. Participants initially completed a medical questionnaire to document any cardiovascular symptoms, family history of SCD or other cardiovascular history.

A longitudinal study design was employed and data were acquired in a resting state during four separate testing sessions; (1) End of pre-season (ENDPRE; Training period before the start of the competitive season) (2) mid-season (MIDCOMP; Middle of competitive season) (3) end-season (ENDCOMP; End of competitive season) and (4) end off-season (ENDOFF: End of the off-season with data collected on athlete return to the club after the end of season break.

All clinical data were analysed and reported by a sports cardiologist and further evaluation if necessary excluded underlying cardiac disease in all participants. All testing sessions were carried out using identical protocols. Retrospective collection of individual training data allowed for changes in workload to be compared with any changes in cardiac data. Training load was calculated by training session duration (minutes) multiplied by athletes’ rating of perceived exertion (RPE) for that session using the Borg category ratio scale (CR10) with rating ranges from 0 to 10 [24] with data collection verified by club science and medical staff. Load was expressed as an arbitrary unit (AU) and mean athlete daily training load data for each seasonal time point was calculated.

### Procedures

All participants abstained from exercise training or recreational activity for at least 6 h prior to each data collection session. Height (Seca 217, Hannover, Germany) and body mass (Seca supra 719, Hannover, Germany) measurements were recorded and body surface area (BSA) was calculated as previously described [25]. Resting arterial blood pressure (BP) was assessed using an automated sphygmomanometer (Dinamap 300, GE Medical systems, USA) [22, 23]. A Standard 12-lead ECG was acquired using commercially available equipment (CardioExpress SL6, Spacelabs Healthcare, Washington US) and interpreted using current “International Criteria” for ECG interpretation in athletes [26].

### Echocardiography

#### Ventricular assessment

The Echocardiographic methods used in this study to assess the LV and right ventricle (RV) have been previously described in the cross sectional studies of RFL athletes [22, 23] and included conventional 2D echocardiography and Speckle Tracking Echocardiography (STE) measurements according to appropriate guidelines [27–29]. Echocardiography data was analysed by a single observer.

#### Atrial assessment

The internal linear measurement of the left atrium (LA) (LAd) was made in the parasternal long axis view at end systole. Biplane LA volume (LAVOLes) was measured at end systole from both the apical 4 chamber and two chamber images [27] whilst right atrial (RA) area (RAa) and volume (RAVOLes) were measured from the apical 4 chamber view only [28]. Further static volume measurements were made from both atria at pre-atrial contraction (LAVOLPreA, RAVOLPreA) and at end diastole (LAVOLed, RAVOLed). From these measurements, LA and RA functional volumes were derived as previously described [30, 31] to provide reservoir (maximal filling) (LAVOLres, RAVOLres), conduit (passive filling) (LAVOLcon, RAVOLcon) and booster (active emptying) (LAVOLboo, RAVOLboo) volumes.

### Statistical analysis

Data were collected and managed using REDCAP electronic data capture tools [32]. Statistical analyses were performed using commercially available software package SPSS Version 23.0 for Windows (SPSS, Illinois, USA). Variables were analysed across the four time points using one-way ANOVA with post-hoc Bonferroni assessment. A P < 0.05 was considered statistically significant.

## Results

Twenty RFL athletes (age 23 ± 4 years (range 18–31)) were recruited at baseline. None of the athletes withdrew from the study and hence completed all data collection points. They had a training history of 13.3 ± 3.6 years and were all participating in a structured training protocol as defined by the club. Training schedules varied between pre and in-season (competitive) periods. During a typical pre-season week, the athletes on average were taking part in 5 field training sessions (skills and conditioning) each of 70 min duration, 4 gym sessions (resistance) each of 60 min duration and 2 ‘wrestle’ sessions each of 40 min duration. During a typical in-season week, athletes were taking part in 3 field sessions each of 45 min duration and 2 gym sessions each of 40 min duration and competitive game play. Depending on athlete selection and or substitution, up to 80 min (full game duration) are spent in competitive RFL gameplay per week. Athletes not selected for competition instead performed 2 training sessions (1 gym session of 40 min and 1 field session of 40 min duration).

**Fig. 1:**
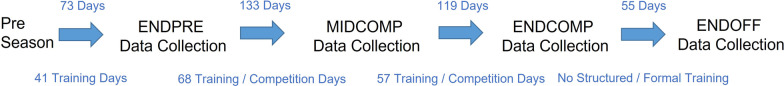
Number of days and number of training/competition days between data collection points

Data was collected as displayed in Fig. 1 which indicates the number of days between testing and the number of training and competition days recorded.

Heart rate, blood pressure and athlete demographic parameters did not vary across the competitive season (P > 0.05; Table 1). Average daily training load was significantly higher at ENDPRE compared to all other assessment points (P < 0.001) with no differences between MIDCOMP and ENDCOMP. All ECGs were normal. Common training related ECG changes; 1st degree AV block, sinus bradycardia and sinus arrhythmia, early repolarisation as well as increased QRS voltage (for LVH or RVH) were largely consistent across the season (Table 2).

**Table 1:** Athlete demographics

	ENDPRE Mean ± SD (Range)	MIDCOMP Mean ± SD (Range)	ENDCOMP Mean ± SD (Range)	ENDOFF Mean ± SD (Range)
Age (years)	23 ± 4 (18–31)	23 ± 4 (18–31)	24 ± 4 (18–31)	24 ± 4 (18–32)
Height (m)	1.84 ± 0.06 (1.70–1.97)	1.84 ± 0.06 (1.70–1.97)	1.84 ± 0.06 (1.70–1.97)	1.84 ± 0.06 (1.70–1.97)
Weight (kg)	98 ± 9 (83–116)	98 ± 9 (84–116)	99 ± 9 (82–116)	99 ± 9 (83–115)
BSA (m^2^)	2.23 + 0.13 (1.98–2.52)	2.24 ± 0.13 (1.99–2.52)	2.24 ± 0.13 (1.98–2.52)	2.25 ± 0.13 (1.98–2.51)
Systolic BP (mmHg)	132 ± 8 (117–155)	135 ± 9 (114–147)	133 ± 11 (117–156)	132 ± 8 (116–146)
Diastolic BP (mmHg)	69 ± 8 (56–81)	73 ± 6 (62–83)	75 ± 7 (64–88)	72 ± 6 (64–89)
HR (Beats min^−1^)	55 ± 9 (42–70)	55 ± 6 (42–62)	56 ± 7 (46–70)	60 ± 8 (46–78)
Average Daily Workload (AU)	715 ± 264*^†^ (83–1316)	323 ± 232* (63–767)	320 ± 242^**†**^ (63–744)	N/A

*Denotes significant difference (P > 0.05) between ENDPRE and MIDCOMP. ^†^denotes significant difference between ENDPRE and ENDCOMP

BSA: body surface area; BP: Blood pressure; HR: Heart rate; AU: Arbitrary Units

**Table 2:** Normal training related ECG changes

Normal training related ECG findings in the athlete	% Athletes ENDPRE	% Athletes MIDCOMP	% Athletes ENDCOMP	% Athletes ENDOFF
Increased QRS Voltage for Left Ventricular Hypertrophy (LVH)	25	20	40	25
Increased QRS Voltage for Right Ventricular Hypertrophy (RVH)	10	5	5	5
Early Repolarisation/ST Segment Elevation	75	85	95	95
Sinus Bradycardia or Sinus Arrhythmia	65	65	75	75
1st Degree AV Block	10	0	0	0

### Left ventricle

Standard 2D echocardiographic LV structural parameters did not differ across the season (P > 0.05; Table 3). With exception of transmitral A wave velocity, which was lower at ENDCOMP compared to ENDOFF (P = 0.028), there were no differences in any standard LV functional index across the season. No between-test differences were observed in LV global ɛ and strain rate (SR) across all three planes of contraction (P > 0.05; Table 4). Apical rotation and twist were higher in ENDPRE compared to MIDCOMP (P = 0.004 and P = 0.027), ENDCOMP (P = 0.002 and P = 0.0009) and ENDOFF (P = 0.019 and P = 0.017).

**Table 3:** Echocardiographic parameters of left ventricular structure and function

	ENDPRE Mean ± SD (Range)	MIDCOMP Mean ± SD (Range)	ENDCOMP Mean ± SD (Range)	ENDOFF Mean ± SD (Range)
LVIDd (mm)	57 ± 3 (50–62)	57 ± 2 (52–60)	56 ± 3 (50–61)	56 ± 3 (51–61)
LVIDd (mm/(m^2^)^0.5^)	38 ± 2 (34–41)	38 ± 1 (35–40)	38 ± 2 (33–40)	37 ± 2 (34–40)
LVIDs (mm)	39 ± 3 (34–44)	39 ± 2 (34–41)	38 ± 2 (33–42)	39 ± 3 (32–43)
LVIDs (mm/(m^2^)^0.5^)	26 ± 2 (23–29)	26 ± 1 (23–29)	26 ± 2 (22–30)	26 ± 2 (21–31)
MWT (mm)	9 ± 1 (8–10)	9 ± 1 (8–10)	9 ± 1 (8–10)	9 ± 1 (8–10)
Max WT (mm)	10 ± 1 (9–11)	9 ± 1 (9–11)	9 ± 1 (8–11)	10 + 1 (8–11)
Max WT (mm/(m^2^)^0.5^)	6.4 ± 0.4 (5.9–7.3)	6.2 ± 0.4 (5.7–7.3)	6.2 ± 0.4 (5.3–7.2)	6.3 ± 0.5 (5.1–7.2)
RWT	0.32 ± 0.03 (0.26–0.37)	0.32 ± 0.03 (0.25–0.37)	0.32 ± 0.03 (0.27–0.40)	0.32 ± 0.04 (0.26–0.38)
LV Mass (g)	195 ± 28 (146–253)	192 ± 24 (135–226)	194 ± 22 (140–226)	188 ± 18 (159–226)
LV Mass (g/(m^2^)^2.7^)	38 ± 5 (29–48)	37 ± 5 (27–44)	38 ± 5 (28–45)	37 ± 4 (31–46)
LV mass (g/m^2^)	87 ± 10 (68–106)	86 ± 9 (64–99)	87 ± 8 (65–97)	84 ± 8 (75–107)
LV Length (mm)	93 ± 4 (83–102)	95 ± 4 (86–102)	95 ± 4 (89–105)	96 ± 3 (87–103)
LVEDV (ml)	141 ± 21 (101–179)	140 ± 22 (104–184)	146 ± 19 (113–179)	148 ± 19 (115–181)
LVEDV (ml/(m^2^)^1.5^))	42 ± 6 (30–57)	42 ± 6 (33–54)	44 ± 5 (35–56)	44 ± 5 (35–58)
LVESV (ml)	61 ± 14 (44–87)	61 ± 11 (44–79)	65 ± 9 (49–82)	65 ± 11 (48–85)
LVESV (ml/(m^2^)^1.5^)	18 ± 4 (11–28)	18 ± 3 (14–26)	19 ± 3 (13–28)	19 ± 3 (15–27)
SV (ml)	80 ± 12 (57–104)	79 ± 13 (54–112)	81 ± 13 (57–112)	83 ± 13 (67–122)
EF (%)	57 ± 5 (48–67)	57 ± 4 (51–67)	55 ± 4 (49–63)	56 ± 4 (48–67)
E Velocity (m/s)	0.83 ± 0.12 (0.52–1.00)	0.78 ± 0.11 (0.55–0.92)	0.79 ± 0.14 (0.58–1.01)	0.82 ± 0.15 (0.46–1.08)
A Velocity (m/s)	0.40 ± 0.07 (0.32–0.55)	0.36 ± 0.08 (0.22–0.56)	0.35 ± 0.07^**§**^ (0.20–0.50)	0.43 ± 0.13^**§**^ (0.25–0.81)
E:A Ratio	2.15 ± 0.46 (1.37–2.78)	2.24 ± 0.61 (1.45–3.82)	2.34 ± 0.60 (1.33–3.65)	2.02 ± 0.61 (0.85–3.16)
Medial S' (cm/s)	9 ± 1 (8–11)	9 ± 1 (8–12)	9 ± 1 (7–12)	9 ± 1 (7–11)
Medial E' (cm/s)	13 ± 2 (10–18)	13 ± 1 (10–15)	14 ± 3 (10–18)	14 ± 2 (10–18)
Medial A' (cm/s)	7 ± 1 (4–9)	7 ± 2 (4–12)	7 ± 1 (3–9)	7 ± 2 (4–11)
Lateral S' (cm/s)	12 ± 2 (9–17)	11 ± 2 (8–14)	11 ± 2 (8–15)	12 ± 2 (8–15)
Lateral E' (cm/s)	19 ± 3 (11–25)	19 ± 3 (12–24)	19 ± 3 (14–24)	18 ± 3 (14–25)
Lateral A' (cm/s)	6 ± 1 (4–9)	6 ± 2 (3–10)	6 ± 2 (4–10)	6 ± 1 (4–9)
Average S' (cm/s)	10 ± 1 (9–14)	10 ± 1 (8–12)	10 ± 1 (8–14)	10 ± 1 (9–13)
Average E' (cm/s)	16 ± 2 (12–20)	16 ± 2 (13–19)	17 ± 2 (13–21)	16 ± 2 (13–19)
Average A' (cm/s)	7 ± 1 (4–9)	6 ± 2 (4–11)	6 ± 1 (4–9)	7 ± 1 (5–10)
Average S' ((cm/s)/cm)	1.10 ± 0.12 (0.96–1.41)	1.03 ± 0.13 (0.82–1.28)	1.06 ± 0.14 (0.77–1.36)	1.09 ± 0.15 (0.89–1.38)
Average E' ((cm/s)/cm)	1.74 ± 0.22 (1.29–2.22)	1.65 ± 0.21 (1.37–2.09)	1.74 ± 0.24 (1.37–2.13)	1.66 ± 0.19 (1.38–1.98)
Average A' ((cm/s)/cm)	0.70 ± 0.11 (0.42–0.89)	0.68 ± 0.20 (0.36–1.22)	0.67 ± 0.15 (0.41–0.93)	0.71 ± 0.13 (0.46–1.01)
Average E/E'	5 ± 1 (4–6)	5 ± 1 (4–7)	5 ± 1 (4–6)	5 ± 1 (3–7)

SD: standard deviation; ^§^ denotes P < 0.05 between ENDCOMP and ENDOFF; LVIDd: left ventricular internal dimension (diastolic); LVIDs: left ventricular internal dimension (systolic); WT: wall thickness; MWT: mean wall thickness; RWT: relative wall thickness; SV: stroke volume; EF: ejection fraction; LVEDV: left ventricular end diastolic volume; LVESV: left ventricular end systolic volume; E: early diastolic velocity; A: late diastolic velocity; S': systolic myocardial velocity; E': early diastolic myocardial velocity; A': late diastolic myocardial velocity

**Table 4:** Global left ventricular ɛ, SR and twist

	ENDPRE Mean ± SD (Range)	MIDCOMP Mean ± SD (Range)	ENDCOMP Mean ± SD (Range)	ENDOFF Mean ± SD (Range)
LV Longitudinal				
Global ɛ (%)	− 19.2 ± 2.2 (− 16.3 to − 23.7)	− 19.5 ± 1.3 (− 16.5 to − 21.4)	− 19.4 ± 1.4 (− 16.0 to–21.6)	− 19.4 ± 1.2 (− 17.5 to − 21.6)
Time to Peak ɛ (s)	0.37 ± 0.03 (0.32–0.43)	0.37 ± 0.03 (0.33–0.41)	0.38 ± 0.02 (0.33–0.41)	0.37 ± 0.03 (0.33–0.44)
SRS (s^−1^)	− 0.94 ± 0.13 (− 0.72 to − 1.31)	− 0.96 ± 0.10 (− 0.81 to − 1.19)	− 0.93 ± 0.08 (− 0.80 to − 1.12)	− 0.94 ± 0.09 (− 0.69 to − 1.12)
SRE (s^−1^)	1.36 ± 0.18 (1.01–1.63)	1.43 ± 0.14 (1.20–1.71)	1.44 ± 0.14 (1.11–1.66)	1.40 ± 0.15 (1.09–1.62)
SRA (s^−1^)	0.56 ± 0.10 (0.37–0.80)	0.58 ± 0.14 (0.36–0.93)	0.55 ± 0.09 (0.36–0.75)	0.59 ± 0.09 (0.41–0.79)
LV Circumferential				
Global ɛ (%)	− 19.1 ± 2.0 (− 16.6 to − 23.3)	− 18.3 ± 1.4 (− 16.1 to − 21.2)	− 18.1 ± 2.1 (− 14.4 to − 21.4)	− 17.5 ± 2.09 (− 14.0 to − 20.4)
Time to Peak ɛ (s)	0.37 ± 0.02 (0.31–0.40	0.37 ± 0.03 (0.32–0.42)	0.39 ± 0.04 (0.33–0.48)	0.38 ± 0.03 (0.32–0.44)
SRS (s^−1^)	− 1.08 ± 0.13 (− 0.90 to − 1.34)	− 1.04 ± 0.13 (− 0.84 to–1.41)	− 1.01 ± 0.11 (− 0.82 to − 1.23)	− 0.98 ± 0.13 (− 0.78 to − 1.19)
SRE (s^−1^)	1.56 ± 0.31 (0.86–2.34)	1.46 ± 0.24 (1.02–1.94)	1.42 ± 0.19 (0.98–1.72)	1.36 ± 0.23 (0.97–1.95)
SRA (s^−1^)	0.44 ± 0.14 (0.29–0.77)	0.38 ± 0.11 (0.22–0.72)	0.37 ± 0.11 (0.19–0.61)	0.40 ± 0.12 (0.18–0.59)
LV Radial				
Global ɛ (%)	46.5 ± 12.8 (27.1–70.2)	49.7 ± 10.3 (34.6–68.4)	51.5 ± 11.5 (31.7–76.7)	45.6 ± 10.9 (28.3–75.0)
Time to Peak ɛ (s)	0.41 ± 0.03 (0.34–0.48)	0.42 ± 0.03 (0.36–0.47)	0.42 ± 0.04 (0.35–0.49)	0.41 ± 0.04 (0.36–0.49)
SRS (s^−1^)	1.51 ± 0.23 (1.11–1.98)	1.55 ± 0.24 (1.06–2.05)	1.60 ± 0.26 (1.29–2.40)	1.54 ± 0.22 (1.22–1.97)
SRE (s^−1^)	− 2.05 ± 0.61 (− 0.99 to − 4.08)	− 2.04 ± 0.33 (− 1.46 to–2.86)	− 2.08 ± 0.47 (− 1.29 to − 2.96)	− 1.97 ± 0.33 (− 1.41 to − 2.59)
SRA (s^−1^)	− 0.85 ± 0.26 (− 0.53 to − 1.30)	− 0.96 ± 0.42 (− 1.74 to–0.17)	− 0.93 ± 0.36 (− 0.42 to − 1.92)	− 0.90 ± 0.25 (− 0.47 to − 1.50)
LV rotation				
Basal rotation (^o^)	− 7.08 ± 3.05 (− 0.29 to − 11.7)	− 6.81 ± 2.50 (− 3.18 to − 13.42)	− 6.78 ± 3.22 (− 0.08 to − 13.75)	− 6.29 ± 1.82 (− 2.97 to − 8.61)
Apical rotation (^o^)	9.83 ± 3.95*^†∫^ (3.35–16.79)	6.13 ± 2.84* (1.88–11.59)	5.84 ± 3.15^**†**^ (0.79–11.52)	6.60 ± 3.07^**∫**^ (2.09–13.98)
Twist (^o^)	16.55 ± 4.71*^†∫^ (6.49–22.63)	12.62 ± 3.97* (3.66–19.9)	12.12 ± 4.53^**†**^ (5.55–19.51)	12.35 ± 3.45^**∫**^ (7.38–19.43)

*Denotes P < 0.05 between ENDPRE and MIDCOMP, ^†^ denotes P < 0.05 between ENDPRE and ENDCOMP, ^∫^ denotes P < 0.05 between ENDPRE and ENDOFF; ɛ: strain; SRS: systolic strain rate; SRE: early diastolic strain rate; SRA: late diastolic strain rate

### Right ventricle and atria

No differences were observed across the season for any RV structural or functional parameters (P > 0.05; Table 5). There were no differences in the left and right atrial parameters at any data collection point (Table 6).

**Table 5:** Structural and functional parameters of the right ventricle

	ENDPRE Mean ± SD (Range)	MIDCOMP Mean ± SD (Range)	ENDCOMP Mean ± SD (Range)	ENDOFF Mean ± SD (Range)
RVOT_PLAX_ (mm)	33 ± 3 (28–41)	34 ± 4 (27–42)	33 ± 3 (29–40)	33 ± 3 (27–42)
RVOT_PLAX_ (mm/(m^2^)^0.5^))	22 ± 2 (19–27)	22 ± 2 (18–28)	22 ± 2 (19–26)	22 ± 2 (18–27)
RVOT_1_ (mm)	34 ± 3 (28–42)	34 ± 3 (27–40)	35 ± 3 (28–42)	35 ± 3 (27–41)
RVOT_1_ (mm/(m^2^)^0.5^))	23 ± 2 (19–28)	23 ± 2 (18–26)	23 ± 2 (19–28)	23 ± 2 (18–27)
RVOT_2_ (mm)	28 ± 3 (23–35)	27 ± 2 (24–33)	27 ± 2 (21–30)	27 ± 2 (22–31)
RVOT_2_ (mm/(m^2^)^0.5^))	19 ± 2 (16–24)	18 ± 1 (16–21)	18 ± 2 (14–20)	18 ± 2 (14–21)
RVD_1_ (mm)	47 ± 4 (39–58)	48 ± 4 (41–54)	48 ± 3 (41–52)	46 ± 3 (40–52)
RVD_1_ (mm/(m^2^)^0.5^))	32 ± 3 (26–38)	32 ± 3 (27–37)	32 ± 2 (28–37)	31 ± 2 (27–35)
RVD_2_ (mm)	37 ± 3 (31–45)	37 ± 3 (32–42)	36 ± 3 (30–44)	35 ± 2 (32–40)
RVD_2_ (mm/(m^2^)^0.5^))	24 ± 2 (21–30)	25 ± 2 (21–28)	24 ± 2 (20–30)	24 ± 1 (21–26)
RVD_3_ (mm)	91 ± 6 (83–102)	93 ± 4 (87–101)	92 ± 5 (82–102)	92 ± 6 (83–103)
RVD_3_ (mm/(m^2^)^0.5^))	61 ± 4 (55–70)	62 ± 3 (56–66)	61 ± 3 (54–67)	61 ± 3 (56–67)
RVDa (cm^2^)	30 ± 3 (23–37)	29 ± 3 (24–36)	29 ± 3 (23–32)	30 ± 3 (23–36)
RVDa (cm^2^/m^2^)	13 ± 2 (11–16)	13 ± 1 (11–16)	13 ± 1 (11–15)	13 ± 1 (10–15)
RVSa (cm^2^)	17 ± 2 (14–20)	16 ± 2 (13–21)	16 ± 2 (11–19)	17 ± 2 (13–20)
RVSa (cm^2^/m^2^)	7 ± 1 (6–9)	7 ± 1 (6–9)	7 ± 1 (5–9)	8 ± 1 (6–9)
TAPSE (mm)	23 ± 2 (19–27)	23 ± 3 (19–29)	24 ± 3 (20–28)	24 ± 3 (20–29)
RV:LV Ratio	0.89 ± 0.06 (0.77–1.00)	0.90 ± 0.07 (0.75–1.00)	0.90 ± 0.05 (0.78–1.00)	0.89 ± 0.06 (0.78–1.00)
RVFAC (%)	45 ± 5 (38–53)	44 ± 5 (36–54)	44 ± 4 (36–54)	43 ± 5 (36–52)
RVS' (cm/s)	13 ± 2 (11–17)	14 ± 2 (11–17)	14 ± 2 (11–17)	14 ± 2 (12–18)
RVS' ((cm/s)/cm)	1.46 ± 0.21 (1.09–1.98)	1.48 ± 0.16 (1.20–1.82)	1.54 ± 0.20 (1.26–1.89)	1.56 ± 0.15 (1.25–1.83)
RVE' (cm/s)	15 ± 3 (9–21)	16 ± 3 (12–23)	16 ± 3 (12–25)	17 ± 4 (11–28)
RVE' ((cm/s)/cm)	1.63 ± 0.35 (1.02–2.38)	1.71 ± 0.32 (1.22–2.47)	1.75 ± 0.39 (1.43–3.01)	1.82 ± 0.43 (1.22–3.15)
RVA' (cm/s)	9 ± 2 (6–15)	9 ± 3 (4–15)	10 ± 2 (6–14)	10 ± 2 (7–13)
RVA' ((cm/s)/cm)	1.02 ± 0.27 (0.59–1.51)	1.01 ± 0.29 (0.45–1.58)	1.08 ± 0.22 (0.63–1.52)	1.07 ± 0.18 (0.73–1.44)
RVɛ (%)	− 26.4 ± 3.4 (− 21.0 to − 34.2)	− 27.4 ± 2.82 (− 21.6 to − 32.3)	− 26.9 ± 2.70 (− 21.6 to − 30.9)	− 27.9 ± 2.61 (− 24.3 to − 33.0)
Time to Peak RV ɛ (s)	0.38 ± 0.03 (0.33–0.43)	0.38 ± 0.03 (0.33–0.44)	0.39 ± 0.02 (0.35–0.43)	0.38 ± 0.03 (0.33–0.45)
RVSRS (s^−1^)	− 1.31 ± 0.19 (− 0.86 to − 1.67)	− 1.36 ± 0.18 (− 1.10 to − 1.85)	− 1.33 ± 0.16 (− 0.99 to − 1.69)	− 1.36 ± 0.17 (− 1.09 to − 1.89)
RVSRE (s^−1^)	1.59 ± 0.32 (1.08–2.28)	1.56 ± 0.31 (0.95–2.02)	1.56 ± 0.29 (1.07–2.19)	1.60 ± 0.33 (1.11–2.42)
RVSRA (s^−1^)	0.80 ± 0.18 (0.49–1.20)	0.85 ± 0.18 (0.43–1.16)	0.83 ± 0.25 (0.34–1.31)	0.88 ± 0.27 (0.40–1.50)

RVOT_PLAX**:**_ Right ventricular outflow tract at parasternal long axis; RVOT_1_: Right ventricular outflow tract (proximal); RVOT_2_: Right ventricular outflow tract (distal); RVD_1_: Right ventricular dimension (basal); RVD_2_: Right ventricular dimension (mid); RVD_3_: Right ventricular length; RVDa: Right ventricular diastolic area; RVSa: Right ventricular systolic area; TAPSE: Tricuspid annular plane systolic excursion; RV:LV ratio: Right ventricular to left ventricular ratio; RVFAC: Right ventricular fractional area change; RVS’: RV TDI lateral systolic myocardial velocity; RVE’: RV TDI early diastolic myocardial velocity; RVA’: RV TDI late diastolic myocardial velocity. RV ɛ: Right ventricular longitudinal strain; RVSRS: Right ventricular systolic strain rate, RVSRE: Right ventricular early diastolic strain rate; RVSRA: Right ventricular late diastolic strain rate

**Table 6:** Echocardiographic parameters of the atria

	ENDPRE Mean ± SD (Range)	MIDCOMP Mean ± SD (Range)	ENDCOMP Mean ± SD (Range)	ENDOFF Mean ± SD (Range)
LAd (mm)	39 ± 3 (33–45)	38 ± 2 (32–42)	39 ± 3 (31–44)	38 ± 3 (33–43)
LAd (mm/(m^2^)^0.5^))	26 ± 2 (23–30)	26 ± 1 (23–29)	26 ± 2 (21–29)	26 ± 1 (23–27)
LAVOLes (ml)	62 ± 11 (45–80)	63 ± 13 (42–83)	66 ± 12 (48–90)	66 ± 11 (44–87)
LAVOLes (ml/(m^2^)^1.5^))	19 ± 4 (14–25)	19 ± 4 (13–27)	20 ± 3 (15–25)	19 ± 3 (14–25)
LAVOLpreA) (ml)	38 ± 7 (26–54)	40 ± 8 (25–51)	41 ± 7 (23–57)	40 ± 8 (26–51)
LAVOLpre A index (ml/(m^2^)^1.5^))	11 ± 2 (8–17)	12 ± 2 (8–16)	12 ± 2 (7–15)	12 ± 2 (8 − 17)
LAVOLed (ml)	26 ± 6 (15–35)	27 ± 7 (16–39)	28 ± 5 (19–41)	27 ± 6 (16–37)
LAVOLed (ml/(m^2^)^1.5^))	8 ± 2 (4–12)	8 ± 2 (5–12)	8 ± 1 (6–10)	8 ± 1 (5–10)
LAVOLres(ml)	37 ± 8 (24–49)	36 ± 9 (23–57)	38 ± 10 (26–56)	39 ± 8 (25–56)
LAVOLcon(ml)	43 ± 12 (13–62)	44 ± 15 (17–68)	43 ± 13 (23–69)	44 ± 11 (24–72)
LAVOLboo (ml)	12 ± 3 (9–22)	13 ± 4 (8–25)	13 ± 4 (4–18)	13 ± 4 (7–20)
RAa (cm^2^)	21 ± 2 (17–24)	19 ± 2 (15–22)	20 ± 2 (15–24)	20 ± 3 (15–25)
RAa (cm^2^/m^2^)	9 ± 1 (7–11)	9 ± 1 (6–10)	9 ± 1 (7–10)	9 ± 1 (7–12)
RAVOLes(ml)	67 ± 10 (48–82)	59 ± 12 (34–81)	68 ± 13 (39–94)	65 ± 13 (36–91)
RAVOLes (ml/(m^2^)^1.5^))	20 ± 3 (14–27)	18 ± 4 (9–24)	20 ± 4 (12–27)	19 ± 4 (11–28)
RAVOLpreA (ml)	45 ± 9 (30–62)	42 ± 10 (24–65)	44 ± 9 (26–65)	42 ± 11 (20–64)
RAVOLpre A Index (ml/(m^2^)^1.5^))	14 ± 3 (10–19)	12 ± 3 (6–18)	13 ± 2 (8–18)	12 ± 3 (6–20)
RAVOLed (ml)	33 ± 9 (20–49)	30 ± 8 (17–43)	33 ± 7 (20–46)	31 ± 8 (16–48)
RAVOLed (ml/(m^2^)^1.5^))	10 ± 3 (5–14)	9 ± 2 (4–13)	10 ± 2 (6–13)	9 ± 2 (5–14)
RAVOLres (ml)	34 ± 7 (22–47)	29 ± 8 (17–45)	34 ± 9 (19–48)	34 ± 8 (20–48)
RAVOLcon (ml)	46 ± 11 (24–74)	50 ± 15 (19–83)	47 ± 13 (23–67)	49 ± 16 (25–93)
RAVOLboo (ml)	13 ± 5 (5–22)	12 ± 4 (7–25)	11 ± 4 (4–19)	11 ± 5 (4–22)

LAd: Left atrial internal diameter; LAVOLes: Left atrial end systolic volume; LAVOLpreA: Left atrial volume pre-atrial contraction; LAVOLed: Left atrial end diastolic volume; LAVOLres: Left atrial reservoir volume; LAVOLcon: Left atrial conduit volume; LAVOLboo: Left atrial booster volume. RAa: Right atrial area, RAVOLes: Right atrial end systolic volume; RAVOLpreA: Right atrial volume pre-atrial contraction; RAVOLed: Right atrial end diastolic volume; RAVOLres: Right atrial reservoir volume; RAVOLcon: Right atrial conduit volume; RAVOLboo: Right atrial booster volume

## Discussion

### Training related ECG changes

Training-related ECG changes are common in elite trained athletes [26] and were noted in this study. Small variance in the number of these changes within the RFL season are likely due to individual variability in training and/or resting status and did not impact on clinical decision making.

### Cardiac structure

In this elite group of RFL athletes there was no significant variation in biventricular structure across the season despite significant differences in training workload. This is in contrast to previous longitudinal in endurance cyclists where a reduction in LV wall thickness was observed in the resting season [9] and an increased LVIDd with reduced LV wall thickness associated with participation in successive Tours de France [10]. Despite differences in sporting discipline, modern professional athletes are chronically trained and a short off-season may not result in any overt detraining effect which, in part, may explain the lack of any significant structural changes seen in this study.

Few studies have analysed seasonal variation in RV structure although RV dilatation was reported in endurance athletes (university rowers) after 90 days of training when compared to baseline assessment [8]. The lack of change in RV data in the current study may reflect a consistently high training load, which may have been a confounding variable in the rower study [33] as well as recent RV assessment [34] in basketball and volleyball players. In the latter study training volume was not defined making between study comparisons challenging. Alongside this, there was a lack of any change in atrial morphology/remodelling as indicated by maximum LA volume (LAVOLes) and maximum RA area (RAa) parameters across the season. We can, therefore, speculate that seasonal variation in training load in elite RFL athletes does not impact on data analysis in the PCS setting.

### Cardiac function

In a previous cross sectional study of RFL athletes, LV ɛ was found to be within published normal limits albeit lower than in controls [22]. Similarly a study by Caselli et al. [35] found normal, but lower LV ɛ in all athletes irrespective of sporting classification. Despite evidence of training load variation, there were no differences in standard systolic functional parameters including global LV ɛ and SR in athletes across the RFL season. In a previous longitudinal study [36] LV ɛ was found to be slightly higher with associated changes in morphology after an 18 weeks training programme in soccer, volleyball and basketball athletes. Data from other longitudinal studies [12, 18, 19, 33] are equivocal but predominantly focus on athletes involved in endurance sports where sessional training volumes are often higher. This makes drawing direct comparisons to RFL athletes challenging, however these studies highlight the importance of training type on short-term cardiac adaptation.

In the current study apical rotation and twist were higher at ENDPRE compared to MIDCOMP, ENDCOMP and ENDOFF, which corresponds to the period of highest training workload. Interestingly, an increase in apical rotation and twist was observed at ENDPRE. This may be explained by the acute, high intensity training load increase in pre-season training (daily workload was more than double than that seen at MIDCOMP and ENDCOMP) that may follow a small deconditioning effect occurring over the off-season. This suggests an increased sensitivity of these functional indices to short-term training load variation [12]. Despite different sporting disciplines, lower apical rotation and LV twist have been previously reported in chronically trained cyclists [37] and RFL athletes [22] in comparison to controls. This has been proposed to be a normal physiological adaptive response of the myocardium to training providing a potential mechanism for development of an adequate contractile reserve [37]. In contrast however, LV rotation and twist measurements did not change in national and international soccer, basketball and volleyball players who were assessed pre and post 18 weeks of intensive training [36], however, again detailed training loads were not defined in this study.

In the current study, the only standard functional index to demonstrate statistically significant variation across the season was late diastolic flow velocity (A). This was higher at the ENDOFF compared to ENDCOMP assessments, which may be related to a detraining effect on the atrial contribution to LV filling. The change in this index was small, however, and was not different between ENDOFF and ENDPRE. Variable reports of changes in diastolic function during longitudinal studies exist, ranging from increases in diastolic function in endurance athletes [8, 12] to decreases in strength athletes [8] to no significant difference in soccer players [14]. A relationship between the change in systolic twist and the subsequent diastolic untwist, to the observed changes in trans-mitral late diastolic velocity cannot be excluded, although a potential mechanism remains difficult to ascertain. The clinical and PCS implications of this isolated finding are likely limited.

Atrial morphology and function are dynamic in athletes and variation over the competitive season in professional soccer players has been observed in response to incremental training loads [17]. Increased reservoir and conduit volumes were reported although a stable active volume remained [17]. In contrast, no functional changes in the atria were observed in the current study as the reservoir, conduit and booster volumes remained consistent throughout the season. Alongside normal systolic and diastolic function presented here, atrial dilatation likely represents normal physiological remodelling. The functional assessment of the atria is nevertheless important for differential diagnosis of physiology and pathology as the dilatation of the atria in athletes may represent normal physiological remodelling but may also represent, for example, LA dysfunction as seen in hypertrophic cardiomyopathy and hypertension [18, 38]. With hypertrophic cardiomyopathy there is evidence of impairment in both reservoir volume and emptying fraction in comparison to athletes and controls [38]. A reduction in reservoir function should always raise the suspicion of a cardiac disorder [15].

No seasonal changes in any RV functional index were observed. This is consistent with global RV functional data in basketball and volleyball players [34]. Conversely, increased RV systolic (RVFAC and TDI) and diastolic function (TDI) was reported in university rowers after 90 days of training [8] and enhanced apical RV ɛ was observed in basketball and volleyball players at pre-season [34]. Variation between findings across studies may again be related to differences in training load variation.

### Summary of key findings

Training related ECG changes were common but largely consistent across testing sessions. There were no differences in conventional structural or functional indices with exception of late diastolic filling velocity, despite considerable variations in training load, across testing sessions. There was a higher degree of apical rotation and twist at ENDPRE when training load was highest.

### Implications

The mechanical changes recorded by STE in this study would not have been recorded in serial cardiac assessments of these athletes by conventional 2D echocardiography alone. Given that, prolonged and reduced LV twist has been reported in cardiomyopathies [39] this study indicates the potential clinical benefit of twist and, therefore, untwist data in the PCS setting. Despite changes in twist, overall cardiac function is normal in RFL athletes. In-exercise echocardiographic studies measuring both functional 2D and STE parameters and their response to exercise at seasonal time points could provide further insight for PCS as well as mechanisms of exercise adaptation in RFL athletes.

## Limitations

A small sample size was used and only male RFL athletes were included in the study, therefore, this data may not be representative of athletes of other sporting disciplines or gender. Observations and assessment was across one competitive season and future studies should focus on longer term follow up. The time periods between data collection points were variable and may have impacted upon the magnitude of changes or lack of changes observed. In addition, despite no formal group training sessions, individual training data was not collected from athletes during the off season. In-exercise echocardiographic studies of RFL athletes may help to validate the functional changes identified by STE.

## Conclusions

Standard ECG and 2D echocardiographic assessment in elite RFL athletes does not appear to be affected by seasonal variation in training load which is reassuring for PCS. Higher apical rotation and twist were noted at ENDPRE and this may extend our knowledge of functional ventricular adaptation to exercise in RFL athletes. STE may provide additional information to aid PCS especially in the role of differential diagnosis in a follow-up / secondary care setting.

## Data Availability

The datasets generated and/or analysed during this study are not publicly available in order to protect privacy.
